# The Educational Efficacy of Humane Teaching Methods: A Systematic Review of the Evidence

**DOI:** 10.3390/ani11010114

**Published:** 2021-01-07

**Authors:** Miriam A. Zemanova, Andrew Knight

**Affiliations:** 1 Animalfree Research, Postgasse 15, 3011 Bern, Switzerland; 2 Centre for Compassionate Conservation, University of Technology Sydney, Ultimo, NSW 2007, Australia; 3 Oxford Centre for Animal Ethics, 91 Iffley Road, Oxford OX4 1EG, UK; 4 Centre for Animal Welfare, University of Winchester, Winchester SO22 4NR, UK; andrew.knight@winchester.ac.uk; 5 School of Environment and Science, Nathan Campus, Griffith University, 170 Kessels Rd, Nathan, QLD 4111, Australia

**Keywords:** 3Rs, alternatives, animal use, education, learning outcome, replacement

## Abstract

**Simple Summary:**

Despite the fact that there are currently many humane teaching methods available, harmful animal use in education and training remains widespread among life and health sciences disciplines. The use of humane teaching methods instead is based not only on legal, ethical, and economic factors, but also on evidence that these training techniques are just as efficient or even better in improving knowledge, understanding, and clinical or surgical skills proficiency among students. However, studies systematically comparing the learning outcomes of both harmful animal use and humane teaching methods are more than a decade old, and the evidence needs to be updated. Here, we assess and summarize the currently available studies through the process of a systematic review. We found 50 relevant studies and established that in 90% of studies humane teaching methods were as or more effective than harmful animal use in achieving desired learning outcomes. These results are clear—there is no valid educational reason for continued harmful animal use in education and training.

**Abstract:**

Humane alternatives to harmful educational animal use include ethically-sourced cadavers, models, mannequins, mechanical simulators, videos, computer and virtual reality simulations, and supervised clinical and surgical experiences. In many life and health sciences courses, however, traditional animal use persists, often due to uncertainty about the educational efficacy of humane alternatives. The most recent comprehensive reviews assessing learning outcomes of humane teaching methods, in comparison to harmful animal use, were published more than 10 years ago. Therefore, we aimed to collate and analyse the combined evidence from recent and older studies about the efficacy of humane teaching methods. Using specific search terms, we systematically searched the Web of Science, SCOPUS, and EMBASE databases for relevant educational studies. We extracted information on publication years, the country in which the study was conducted, field, humane teaching methods, form of learning outcome assessment, and the learning outcome of the humane teaching methods, in comparison with harmful animal use. We found 50 relevant studies published from 1968–2020, primarily stemming from the USA, UK, and Canada. Humane teaching methods produced learning outcomes superior (30%), equivalent (60%), or inferior (10%) to those produced by traditional harmful animal use. In conclusion, a wide-spread implementation of humane teaching methods would not only preserve learning outcomes, but may in fact be beneficial for animals, students, educators, and institutions.

## 1. Introduction

Students in life sciences need to learn numerous skills in order to become experts in their field. In some subjects, harmful animal use has been deemed necessary for efficient learning. Animals have been, and continue to be, killed to obtain cadavers and body parts for anatomical dissection, and subjected to invasive experiments to demonstrate scientific concepts within subjects such as physiology, biochemistry, pharmacology, and parasitology. The animals are often killed at the end of these procedures. Within veterinary surgical and clinical skills training, students in many countries have traditionally practiced surgical procedures on healthy animals before killing them via anaesthetic overdose [1].

However, the policies at schools and universities supporting the use of animals were often implemented decades ago [2] and their revision may be long overdue. Furthermore, the growing interest in animal welfare within academic institutions [3] as well as the increasing opposition from the general public against using animals in experimental procedures [4,5] have led to questioning the necessity of continued harmful animal use within education. 

Currently, there are many humane teaching methods available, which can be broadly classified into the following groups [6]: (1) models, mannequins and mechanical simulators, (2) computer and virtual reality simulations, (3) videos, (4) self-experimentation, (5) observational studies, (6) studies on cell lines and organotypic cultures in vitro, (7) ethically-sourced animal cadavers (from animals that have died naturally or in accidents, or been euthanased for genuine medical reasons or severe and intractable behavioural problems), and (8) supervised clinical practice. While humane teaching methods have been shown to be commonly superior to harmful animal use in terms of costs [7,8,9], time [10,11], ethics [12], and psychological impact on students [13,14,15,16,17,18], their implementation is still lagging, as evidenced by the high numbers of animals used for educational purposes, recorded in the annual statistics of animal use for scientific purposes within Europe [19]. One of the obstacles to moving away from the harmful use of animals within education and training might be the perception that humane methods are not as effective in providing the intended learning outcomes [20,21,22]. This, indeed, was the reason most commonly reported to justify animal use, within a review of non-technical summaries of projects using live animals for scientific purposes published within Europe from 2017–2019 [22].

So far, to the authors’ knowledge, there have been no recent, comprehensive reviews of the efficacy of humane teaching methods, with the most recent, large reviews conducted over a decade ago [6,23]. To provide a comprehensive and contemporary review of the evidence in this field, we designed a systematic review to collate, pool, and analyse both recent and older evidence concerning the efficacy of humane teaching methods in comparison to harmful animal use, within life and health sciences education and training. 

## 2. Materials and Methods

We followed the PRISMA guidelines for conducting systematic reviews [24]. We searched three of the largest databases with a complementary coverage of medicine, life sciences, social science, and technology fields [25] to identify the relevant studies published in peer-reviewed journals: Web of Science, SCOPUS, and EMBASE. We used several terms that would most likely capture the relevant educational studies, combined into the following search string: TI (title includes) = ((education* OR training OR teaching OR learning OR student OR school OR skills OR competenc* or curriculum OR pedagog* OR develop* OR demonstration) AND (alternative OR simulat* OR comput* OR online OR dissection OR model* OR virtual OR reality OR anatom* OR physiolog* OR surg*OR veterinary OR medicine OR pharmacolog* OR method OR video OR traditional OR organ OR clay OR interactive OR resource OR laboratory OR cadaver OR clinical OR humane) AND (efficacy OR effective* OR impact OR evaluation OR impression OR benefit* OR drawback* OR outcome* OR value* OR perception OR implementation OR assessment OR need OR comparison OR increase OR decrease OR acquisition OR achievement OR knowledge OR success* OR replace* OR difference OR value OR experience OR performance OR attitude OR effect)) AND AB (abstract includes) = animal. There was no limit on the years considered. The search was conducted on 11 July 2020. The generated list of titles and abstracts was then scanned for relevant studies.

Defined as relevant were studies that presented a comparison of the learning outcome of harmful animal use (i.e., any procedure that does not benefit the animal) and a humane teaching method. When we were not sure if a study was suitable based on its title and abstract, we downloaded the full text to assess the main body of the text. Excluded from further analysis were reviews, opinion pieces, attitude assessments, references without a full text available, and studies describing humane methods without comparison to harmful animal use. For each of the identified relevant studies, we extracted both qualitative and quantitative data. Specifically, we recorded these parameters: (1) year of publication, (2) country where the study was conducted, (3) academic discipline (e.g., veterinary medicine), (4) education level (secondary or tertiary), (5) species used in the harmful animal use, (6) humane teaching method, (7) number of students taught through the humane method, and the total number of students within the study, (8) form of assessment of teaching efficacy, and (9) conclusion about the teaching efficacy of the humane method (superior, equivalent, or inferior learning outcome, in comparison to harmful animal use).

## 3. Results

### 3.1. Search Results and Study Selection

**Figure 1:**
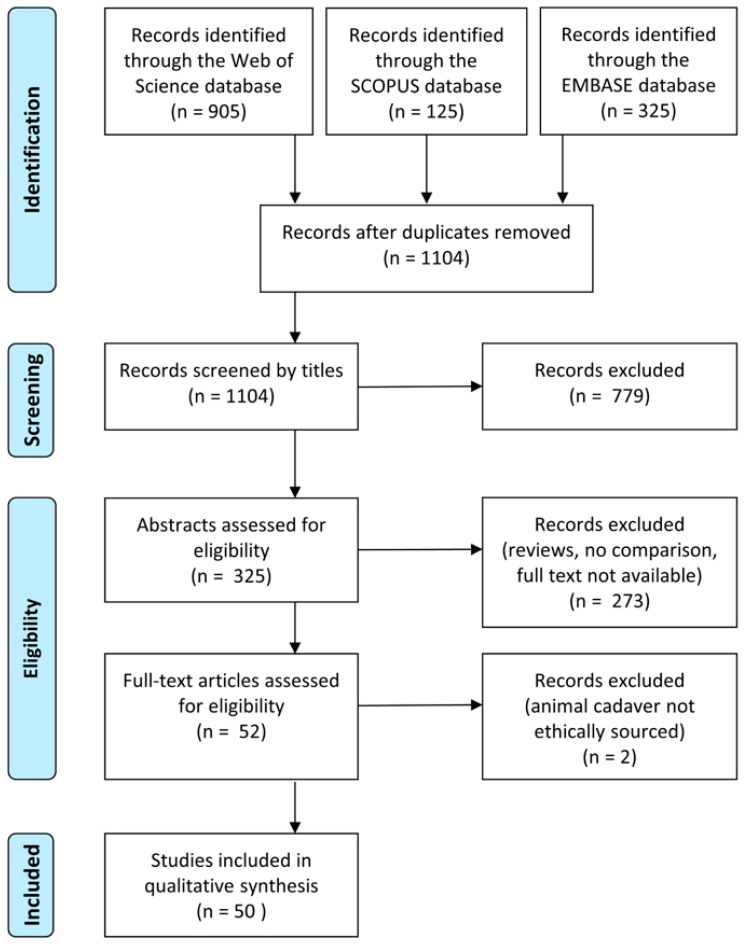
PRISMA literature search flow diagram. The number of studies (n) that were identified, screened, retained, or discarded are shown at each stage of the systematic review process.

**Figure 2:**
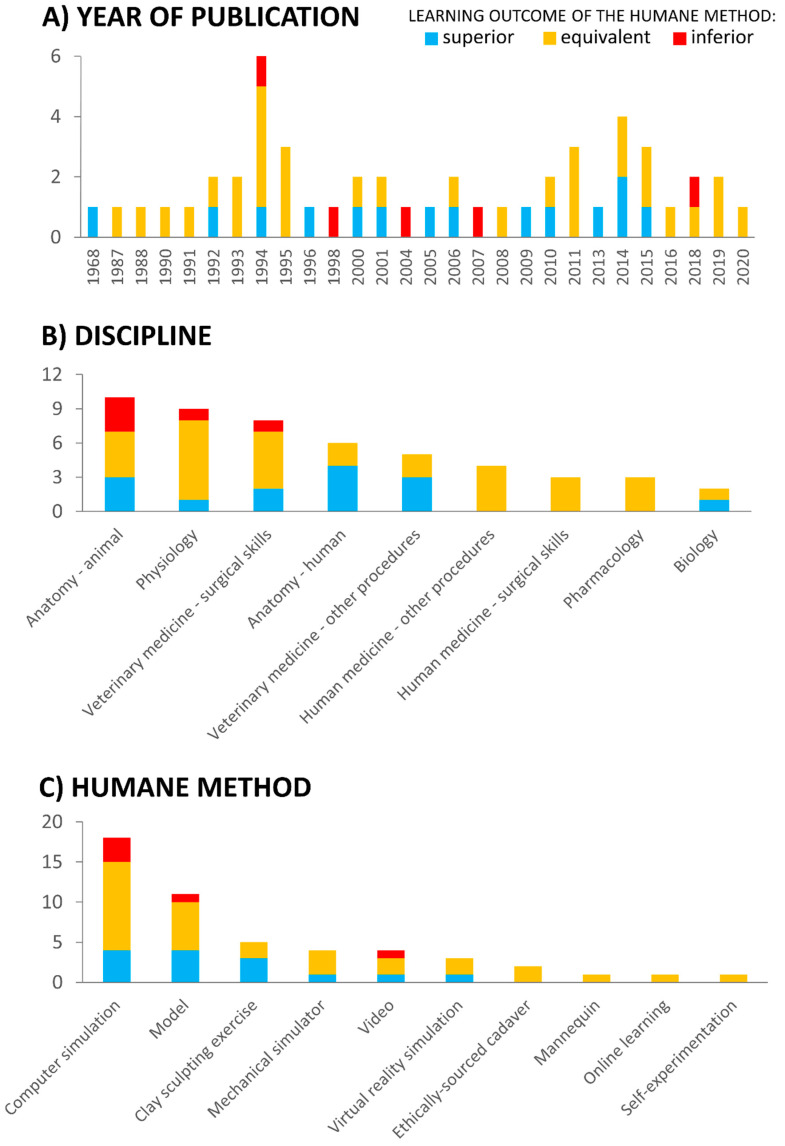
Number of studies comparing learning outcomes of humane teaching method and harmful animal use: (**A**) from 1968–2020, (**B**) grouped by discipline and (**C**) by humane method used. Note: Years with zero publications are not included in (**A**).

In total, we identified 1104 unique references, of which 50 were relevant studies that compared harmful animal use and humane teaching methods (Figure 1). The studies were conducted in 12 different countries, with the majority stemming from the USA (n = 31), followed by the UK (n = 7) and Canada (n = 3; Table 1). The earliest paper identified in our systematic search was published in 1968, and the most recent in 2020, with 16 published in the most recent decade from 2011–2020. The number of articles published per year ranged from 0–6 (Figure 2A; Table 1). The majority of studies focused on tertiary education (n = 44), spanning nine disciplines and sub-disciplines (Figure 2B; Table 1). Highest represented within the 50 relevant studies were articles on teaching animal anatomy, physiology, and surgical skills in veterinary medicine (Figure 2B; Table 1). In terms of animal species, humane methods were primarily compared to the harmful use of dogs (n = 11), frogs (n = 9), rats (n = 8), and pigs (n = 6; Table 1). The number of students using the humane method in studies ranged from 6 to 308. (Table 1). The most common methods of learning outcome assessment were examinations testing knowledge acquired (n = 30) and evaluations of task performance (n = 17; Table 1).

When considered overall, the majority of the studies showed either equivalent (60%; n = 30) or superior (30%; n = 15) efficacy of humane methods in achieving learning outcomes, in comparison to harmful animal use. There also seems to have been a slight improvement in the learning outcomes of humane teaching methods over time. During the last decade, only one study reported an inferior learning outcome in comparison to harmful animal use, compared to two per decade, in each of the previous decades (Figure 2A; Table 1).

### 3.2. Computer and Virtual Reality Simulation

A total of 21 studies compared computer or virtual reality simulation to harmful animal use, making such non-mechanical simulators, when viewed in combination, the most common humane methods (Figure 2C; Table 1). These methods had the same teaching efficacy as harmful animal use in physiology experiments [7,8,10,33,34,35], pharmacology teaching [41,49], animal anatomy demonstration [51,65], teaching of biology concepts [11], veterinary medicine [55], as well as in human medicine surgical skills [58].

Several studies demonstrated superior learning outcomes of humane teaching methods. For instance, in the study by Abutarbush et al. [26], students were trained to pass a nasogastric tube in the horse, and those taught through computer-assisted learning performed the task better. When learning animal anatomy, high school students that used the virtual reality platform V-Frog obtained higher examination scores than those learning through physical frog dissection [52], and a similar result was reported also for E-Rat versus live rat dissection [62]. Computer and virtual reality simulations were shown to produce superior learning outcomes in general biology [56] and physiology teaching [63] as well.

In contrast to the predominant study findings above, three studies reported inferior learning outcomes with computer simulations: fetal pig dissection [54], frog dissection [30], and in neurophysiology laboratory training [67].

### 3.3. Models, Mannequins, and Simulators

The second most common humane teaching methods were models, mannequins, and mechanical simulators (n = 16; Figure 2C; Table 1). These teaching methods resulted in equivalent learning outcomes to those achieved by harmful animal use, when teaching neonatal intubation [27], laparoscopy [9,48], animal anatomy [39], human anatomy [53], general biology [36], surgical skills in veterinary medicine [42,43,44], and training of emergency medical procedures, such as insertion of a chest tube [45,46] and cricothyroidotomy [50].

Superior learning outcomes were reported in three studies in veterinary medical education. Mechanical simulators and mannequins performed better in teaching both surgical [60] and other skills [38,59].

There was one study that reported an inferior learning outcome of the humane teaching method in teaching veterinary surgical skills. Smeak et al. [64] created a hollow organ simulator consisting of a laminated elastic stomach cast housed in a videotape case. In comparison to surgery on live dogs, this simulator did not prepare the students as well for the surgical task. However, the authors identified some inadequacies with the model in the course of the study (sutures pulled out despite appropriate positioning and technique) and discussed the need for reformulation of the simulator material.

### 3.4. Other Humane Teaching Methods

We were able to identify another five humane teaching methods across 14 studies that yielded equivalent or superior learning outcomes (Figure 2C; Table 1). Equivalent learning outcomes were achieved through the use of ethically sourced cadavers in veterinary surgical training [28,61], through self-experimentation with own blood [29], or through video [37] in physiology classes, clay sculpting in human anatomy teaching [32,69], and through an online learning module [70] or video [31] in veterinary medicine.

Superior learning outcomes were reported with the use of a video in teaching earthworm, crayfish, frog, and fish anatomy [40], but use of video resulted in an inferior outcome in a bovine anatomy teaching study [66]. Superior learning outcomes were also observed with clay sculpting. Motoike et al. [57] and Waters et al. [68] reported that clay sculpting was more effective than cat dissection for learning human anatomy. Similarly, Haspel et al. [47] compared clay sculpting and rat dissection in a human anatomy and physiology curriculum. The students learning through the humane method received a higher exam grade at the end of the course.

## 4. Discussion

### 4.1. Teaching Efficacy of Humane Methods

We implemented a systematic review strategy because this provides a powerful tool to identify, collect, evaluate, and summarize research evidence, in a highly objective, transparent, and reproducible manner [71]. The majority of the studies showed either equivalent or superior efficacy of humane methods (jointly, 90%) in achieving learning outcomes, in comparison to harmful animal use. Only five studies (10%)—in the fields of animal anatomy, physiology, and veterinary surgical training—indicated higher efficacy of harmful animal use in comparison to using computer simulations, models, or videos (Figure 2B). The inferior learning outcomes in these cases could be explained by inappropriate application or inadequate design of the humane teaching method. For instance, Cross and Cross [30] compared virtual and live frog dissections. The authors suggested that the poor performance of students taught through the humane method was due to the computer simulation not being extensive enough to cover the advanced knowledge tested in the exam. The only study published within the last decade and reporting inferior learning outcome of the humane teaching method, was by Wang et al. [67]. The authors developed and assessed computer simulation used in neurophysiology laboratory training, which did not perform well. Wang et al. [67] admitted that their program needs to be improved in terms of design to make it “more realistic and practical”. These studies proved that it is essential that humane methods are well designed, in order to achieve an effective application within training [72].

We also noted the slight improvement in the learning outcomes of humane teaching methods over time (Figure 2A). A likely explanation for this temporal trend is the much higher fidelity and efficacy of modern humane teaching methods. For example, computer and virtual reality simulations were by far the most represented humane teaching method (n = 21; Table 1), reflecting the growth and technological advances in this field. Also, the currently available high-fidelity models and patient-specific virtual reality systems are much more realistic and collaborative than the methods from the past [73]. At the same time, in the majority of the studies, even the ‘old’ humane teaching methods were still superior in terms of their teaching efficacy to harmful animal use.

In summary, 90% of the studies reported superior or equivalent efficacy of humane teaching methods. Consequently, while this updated systematic review has identified additional studies since the last major reviews in this field, published in 2007 [6,23], our updated findings are consistent, reporting similar results to these previous reviews, and strengthen their earlier conclusions.

### 4.2. Trends within Disciplines

Studies in veterinary surgical skills or other procedures (n = 13) and animal anatomy (n = 10) were most common, reflecting the highest use of animals within these disciplines. Hence, a transition towards humane teaching methods might be particularly important for veterinary students.

Other highly represented fields within this review were human anatomy and medicine (n = 13). None of these studies showed inferior learning outcomes when using a humane teaching method, in comparison to harmful animal use. Waters et al. [69] pointed out in their study comparing the learning outcomes of clay sculpting and cat dissection that, “If one wants to teach human anatomy, then a specimen that looks like a human (even a human anatomy clay sculpture) is preferable to a nonhuman specimen”. Similarly, in the study by Lombardi et al. [53], students learning via plastic human heart models performed better on an examination, than students learning through dissection of sheeps’ hearts. Some countries and universities have already recognized that it is unnecessary to use animals for teaching human medicine. For instance, all US medical schools had transitioned their curricula to non-animal teaching, by 2016 [74].

### 4.3. Pedagogical Factors Affecting Teaching Efficacy

Many simulators, unlike animals, accurately replicate key aspects of the human body and allow human medical students to repeat clinical skills procedures, or otherwise customize learning experiences towards individual learner needs [52,59,75]. Such repeated practice leads to greater skill retention [27]. Live animal laboratories are also very time-intensive, with the majority requiring several hours to set up, prepare and stabilize animals, conduct procedures, recover or euthanase animals, and to clean and pack down. Humane alternatives are frequently more time-efficient [10,11], freeing student and staff time for additional learning or other academic activities.

Humane alternatives can also positively impact student learning and attitudinal development in less obvious ways. For many students, harmful live animal use, such as that occurring in physiology demonstration or surgical training laboratories, can create high levels of stress. As noted by Gelberg and Gelberg [18], most students are drawn to the veterinary profession because of their strong concern for animals; hence it can be particularly stressful for them to see animals harmed through educational activities, or worse, to be required to personally inflict that harm through required procedures, or by killing animal subjects afterward. Such stress has considerable potential to adversely affect the cognitive processes required for learning. Surveys indicate that veterinary students are often distracted from relevant scientific concepts by the plight of their animals and the necessity of concentrating on maintaining life and appropriate levels of anaesthesia [76].

Some profound and disturbing attitudinal effects may also result. As Martinsen [77] notes of veterinary students, “Animal experiments habituate the students into accepting the instrumental use of animals”. The decreasing awareness of veterinary students of animal sentience (specifically, the hunger, pain, fear, and boredom of dogs, cats, and cows) over the duration of their veterinary courses [78], the decreased likelihood of fourth-year students to provide analgesia when compared to second or third-year students [79], and the inhibition of normal development of moral reasoning ability during the four years of veterinary school [80], have all been revealed within veterinary student cohorts, and described by us elsewhere [76].

These are all desensitization-related phenomena. These are actually psychological adaptations to ‘cognitive dissonance’—a discordance between behaviour and beliefs. The behaviour in this case is harmful educational animal use, and the belief is that animals are sentient and should not be harmed. Humans normally resolve such dissonance, either by altering behaviour, or beliefs. When altering behaviour is not an option—because students fear that refusal to participate could threaten their careers, then beliefs can change, with the result being adverse attitudinal changes toward animals. Arluke and Hafferty [81] demonstrated that learning experiences perceived as morally wrong ultimately lead to desensitization through the use of absolutions justifying the act. The desensitization process is actually seen by some teachers as an educational objective, aimed at better preparing students for the ‘real world’. Medical students, for example, are reportedly “advised to disengage ourselves from our emotions when dealing with patients” [82]. However, ‘compassion fatigue’ is characterized by diminished ability to empathize or feel compassion for others. Adaptations such as these resolve the dissonance, enabling previously caring students to withstand what could otherwise be intolerable psychological stresses resulting from requirements to harm and kill sentient creatures in the absence of overwhelming necessity [17,83]. However, such changes potentially risk future animal patients, when veterinarians affected by these psychological phenomena become less likely to consider animals as sentient, or to provide appropriate analgesia.

### 4.4. Other Advantages of Humane Teaching Methods

Apart from conclusions about teaching efficacy, some of the reviewed studies also highlighted additional advantages of humane methods. Live animals or specimens can be costly to acquire, house, feed, or otherwise maintain, anaesthetise, euthanase, and dispose of in accordance with regulations. After initial investment, humane teaching methods are frequently cheaper than animal use [7,8,9,35,52].

Ethically-sourced cadavers may be obtained from donation programs established within veterinary teaching hospitals or partner veterinary practices. The first cadaver donation program was implemented at the Tufts University School of Veterinary Medicine (now the Cummings School of Veterinary Medicine at Tufts University) in 1996 [84], but many have since been established in veterinary schools within the US and elsewhere. The Tufts programme supplies all cadavers needed for educational purposes, including first year gross anatomy, and clinical skills and medical procedures laboratories. Reported advantages included increased biological diversity between specimens, integration of clinical histories and pathological conditions, financial savings, and elimination of student and faculty objections to the use of purpose-killed animals [84,85].

Body parts, however sourced, may be permanently preserved using a variety of methods [86]. This prevents the need for fresh samples to be regularly preserved, with increased risk of student and staff exposure to highly toxic liquid chemicals and volatile gases. These create significant health risks as well as the potential for legal and financial liability should adverse exposure-related effects result. In the experience of one of us (Knight) and colleagues from leading veterinary schools internationally, recommended safety guidelines such as the use of gloves, gowns, and masks are sometimes met with only partial compliance [6].

### 4.5. Other Types of Humane Teaching Methods

Some types of humane alternatives did not feature in the comparative studies of learning outcomes retrieved in our systematic review, because studies describing them did not compare learning outcomes with those achieved by harmful animal use. However, they may nevertheless offer important learning benefits. Baillie et al. [87,88,89], for example, developed and trialled a Bovine Rectal Palpation Simulator. Rectal palpation facilitates pregnancy diagnosis in cows and is an important clinical procedure in cattle medicine. Instead of using live cows, this simulator uses a software-controlled robotic arm inside the simulated hindquarters of a cow. The arm applies anatomically-appropriate forces to the fingertips of students, depending on their location within the simulation. This is a ‘haptic’ simulator—one applying tactile feedback and simulated kinaesthetic forces. Baillie and colleagues found that veterinary students using the simulator performed better when examining live cows for the first time.

As we have described previously [6], supervised clinical experiences form the most important element of alternative surgical training. After mastering basic manual skills such as suturing and instrument handling using models, students ideally progress to surgical training using ethically-sourced cadavers. Finally they should observe, assist with, and perform surgery under direct one-to-one supervision, on patients that genuinely benefit. In this case, models and cadavers are being used to better prepare students for subsequent live animal use, with likely benefits including reduced surgical and anaesthetic time. As we have noted [6]:

“[clinical and surgical] rotations are more likely to expose students to a higher volume of commonly-encountered conditions … Resultant benefits include greater exposure to the clinical histories, examinations, and presenting signs of cases more directly relevant to new graduates, and to the diagnostic workups and post-operative management of such cases. Surgical participation is normally conducted under close individual supervision, as distinct from the group supervision normally provided during veterinary school surgical laboratories.”

### 4.6. Transitioning toward Humane Teaching Methods

The number and diversity of humane teaching methods have grown significantly in the last decades. Nowadays, the InterNiche Alternatives Database lists approximately 1400 humane teaching methods (http://www.interniche.org/en/alternatives). Considering the availability of humane methods and the evidence-based conclusions affirming their teaching efficacy, one must ask: Why does harmful animal use still persist?

Previous studies have suggested these explanations: (a) awareness about humane teaching methods might be lacking [90], (b) some teachers and instructors could be resistant to change [91,92], (c) there is a requirement of initial investment of money and time when first introducing and implementing a new method [93], or (d) governmental regulation or incentive might be inadequate [94].

These challenges need to be addressed at multiple levels: (1) the training of life and health sciences educators should be designed to increase their awareness about the efficacy of humane teaching methods, (2) exchange of information and best practice strategies among universities should be encouraged, (3) there needs to be more financial support from governmental and international institutions to universities for implementing alternatives, as well as for non-profit organizations that are distributing information about humane teaching methods (e.g., InterNiche, Animalearn), and (4) more stringent enforcement of legislation requiring alternatives to animal use, is necessary.

## 5. Conclusions

This systematic review identified a broad range of studies comparing learning outcomes achieved by traditional harmful animal use, with those achieved by humane teaching methods, including computer and virtual reality simulations, models, mannequins and simulators, videos, cadavers, self-experimentation, clay sculpting exercises, or online learning modules. Overall, 90% of the assessed studies found that humane teaching methods provide superior or equivalent learning outcomes to harmful animal use. The remaining 10% highlighted the importance of the teaching methods being well-designed and appropriately implemented: poor validity was associated with a lack of realism. In conclusion, this systematic review confirmed the findings of previous studies and overwhelmingly demonstrated that harmful animal use for teaching and training is not justified. As new studies continue to be regularly published in this field, and continue to demonstrate the efficacy of humane teaching methods [95], we recommend that this systematic review be repeated, at least every five years.

## Figures and Tables

**Table 1:** Relevant studies identified in the systematic review, comparing the learning outcome of a humane teaching method with harmful animal use.

Study	Country	Discipline	Level	Species	Humane Method	Students: Humane Method (Total)	Assessment of Learning Outcome	Learning Outcome of the Humane method
Abutarbush et al., 2006 [26]	Canada	Veterinary medicine—other procedures	Tertiary	Horse	Computer simulation	27 (52)	Exam; task performance	Superior
Andreatta et al., 2015 [27]	USA	Human medicine—other procedures	Tertiary	Cat	Mannequin	167 (294)	Exam; task performance	Equivalent
Botden et al., 2010 [9]	Nether-lands	Human medicine—surgical skills	Tertiary	Pig	Model	20 (20)	Task performance	Equivalent
Carpenter et al., 1991 [28]	USA	Veterinary medicine—surgical skills	Tertiary	Dog	Ethically sourced cadaver	12 (24)	Task performance	Equivalent
Clarke 1987 [7]	UK	Physiology	Tertiary	Frog	Computer simulation	15 (28)	Exam	Equivalent
Cronholm 2000 [29]	Sweden	Physiology	Tertiary	Rat	Self-experimentation	94 (133)	Exam	Equivalent
Cross and Cross 2004 [30]	USA	Animal anatomy	Secondary	Frog	Computer simulation	38 (74)	Exam	Inferior
Davy et al., 2019 [31]	USA	Veterinary medicine—other procedures	Tertiary	Dog	Video	19 (38)	Task performance	Equivalent
DeHoff et al., 2011 [32]	USA	Human anatomy	Tertiary	Cat	Clay sculpting exercise	88 (193)	Exam	Equivalent
Dewhurst et al., 1988 [33]	UK	Physiology	Tertiary	Frog	Computer simulation	66 (112)	Exam	Equivalent
Dewhurst and Meehan 1993 [34]	UK	Pharmacology	Tertiary	NA	Computer simulation	NA (65)	Exam	Equivalent
Dewhurst et al., 1994 [35]	USA	Physiology	Tertiary	Rat	Computer simulation	6 (14)	Exam	Equivalent
Downie and Meadows 1995 [36]	UK	Animal anatomy	Tertiary	Rat	Model	308 (2913)	Exam	Equivalent
Durand et al., 2019 [37]	Brazil	Physiology	Tertiary	Rat	Video	108 (350)	Exam	Equivalent
Eichel et al., 2013 [38]	Germ-any	Veterinary medicine—other procedures	Tertiary	Horse	Model	12 (24)	Task performance	Superior
Fančovičova and Prokop 2014 [39]	Slovakia	Animal anatomy	Tertiary	Fish, rat	Model	15 (46)	Exam	Equivalent
Fawver et al., 1990 [10]	USA	Physiology	Tertiary	Dog	Computer simulation	18–24 (85)	Exam	Equivalent
Fowler and Brosius 1968 [40]	USA	Animal anatomy	Secondary	Earthworm, crayfish, frog, fish	Video	NA (156)	Exam	Superior
González Guevara et al., 2008 [41]	Colom-bia	Pharmacology	Tertiary	Guinea-pig	Computer simulation	73	Perceived clarity of information	Equivalent
Greenfield et al., 1994 [42]	USA	Veterinary medicine—surgical skills	Tertiary	Dog	Model	36 (36)	Task performance	Equivalent
Greenfield et al., 1995 [43]	USA	Veterinary medicine—surgical skills	Tertiary	Dog	Model	18 (36)	Task performance	Equivalent
Griffon et al., 2000 [44]	UK	Veterinary medicine—surgical skills	Tertiary	Dog	Model	20 (40)	Task performance	Superior
Hall and Andrew 2011 [45]	USA	Human medicine—other procedures	Tertiary	Pig	Mechanical simulator	12 (24)	Task performance	Equivalent
Hall et al., 2014 [46]	USA	Human medicine—other procedures	Tertiary	Pig	Mechanical simulator	23 (101)	Perceived self-efficacy	Equivalent
Haspel et al., 2014 [47]	USA	Human anatomy	Tertiary	Cat, rat	Clay sculpting exercise	304 (747)	Exam	Superior
Heinrich et al., 2006 [48]	Germ-any	Human medicine—surgical skills	Tertiary	Rabbit	Model	6 (12)	Task performance	Equivalent
Hughes 2001 [49]	UK	Pharmacology	Tertiary	Guinea-pig	Computer simulation	66 (112)	Exam	Equivalent
Iverson et al., 2015 [50]	USA	Human medicine—other procedures	Tertiary	Pig	Mechanical simulator	33 (69)	Task performance	Equivalent
Kinzie et al., 1993 [51]	USA	Animal anatomy	Secondary	Frog	Computer simulation	15 (61)	Exam	Equivalent
Lalley et al., 2010 [52]	USA	Animal anatomy	Secondary	Frog	Virtual reality simulation	51 (102)	Exam	Superior
Leathard and Dewhurst 1995 [8]	UK	Physiology	Tertiary	Rat	Computer simulation	70–80 (156)	Exam	Equivalent
Leonard 1992 [11]	USA	Biology	Tertiary	Frog, mouse	Computer simulation	70 (142)	Exam	Equivalent
Lombardi 2014 [53]	USA	Human anatomy	Tertiary	Sheep heart	Model	16 (29)	Exam	Superior
Matthews 1998 [54]	USA	Animal anatomy	Secondary	Pig	Computer simulation	12 (20)	Exam	Inferior
McCool et al., 2020 [55]	USA	Veterinary medicine—surgical skills	Tertiary	Dog	Virtual reality simulation	6 (12)	Task performance	Equivalent
More and Ralph 1992 [56]	USA	Biology	Tertiary	NA	Computer simulation	93 (184)	Exam	Superior
Motoike et al., 2009 [57]	USA	Human anatomy	Tertiary	Cat	Clay sculpting exercise	NA (181)	Exam	Superior
Mouraviev et al., 2016 [58]	USA	Human medicine—surgical skills	Tertiary	Pig	Virtual reality simulation	10–11 (21)	Task performance	Equivalent
Nagel et al., 2015 [59]	Austria	Veterinary medicine—other procedures	Tertiary	Horse	Mechanical simulator	8 (25)	Task performance	Superior
Olsen et al., 1996 [60]	USA	Veterinary medicine—surgical skills	Tertiary	Dog	Model	20 (40)	Task performance	Superior
Pavletic et al., 1994 [61]	USA	Veterinary medicine—surgical skills	Tertiary	Dog	Ethically sourced cadaver	12 (48)	Skills assessment by employers	Equivalent
Predavec 2001 [62]	Austra-lia	Animal anatomy	Tertiary	Rat	Computer simulation	233 (401)	Exam	Superior
Samsel et al., 1994 [63]	USA	Physiology	Tertiary	Dog	Computer simulation	110	Perceived usefulness of the teaching	Superior
Smeak et al., 1994 [64]	USA	Veterinary medicine—surgical skills	Tertiary	Dog	Model	20 (40)	Task performance	Inferior
Strauss and Kinzie 1994 [65]	USA	Animal anatomy	Secondary	Frog	Computer simulation	9 (20)	Exam	Equivalent
Theoret et al., 2007 [66]	Canada	Animal anatomy	Tertiary	Cow	Video	37 (75)	Exam	Inferior
Wang et al., 2018 [67]	China	Physiology	Tertiary	Frog	Computer simulation	18 (23)	Exam	Inferior
Waters et al., 2005 [68]	USA	Human anatomy	Tertiary	Cat	Clay sculpting exercise	60 (136)	Exam	Superior
Waters et al., 2011 [69]	USA	Human anatomy	Tertiary	Cat	Clay sculpting exercise	75 (196)	Exam	Equivalent
Winder et al., 2018 [70]	Canada	Veterinary medicine—other procedures	Tertiary	Cow	Online learning	23 (43)	Task performance	Equivalent

## Data Availability

The data presented in this study are available on request from the corresponding author.
